# Impact of Insecticides on Parasitoids of the Leafminer, *Liriomyza trifolii,* in Pepper in South Texas

**DOI:** 10.1673/031.011.6101

**Published:** 2011-05-17

**Authors:** Ricardo Hernández, Marvin Harris, Tong-Xian Liu

**Affiliations:** ^1^ Department of Entomology, Texas AgriLife Research, Texas A&M University System, 2415 E. Highway 83, Weslaco, TX 78596-8399, USA; ^2^ Department of Entomology, Texas A&M University, 2475 TAMU, College Station, TX 77843-2475, USA; ^3^ Current address: Key Laboratory of Applied Entomology, Northwest A&F University, Yangling, Shaanxi, 712100, China

**Keywords:** conservation biological control, *Neochrysocharis formosa*, *Closterocerus cinctipennis*, *Opius dissitus*, *Cirrospilus variegates*

## Abstract

*Liriomyza* leafminers (Diptera: Agromyzidae) are cosmopolitan, polyphagous pests of horticultural plants and many are resistant to insecticides. Producers in South Texas rely on insecticides as the primary management tool for leafminers, and several compounds are available. The objective of this study is to address the efficacy of these compounds for controlling *Liriomyza* while minimizing their effects against natural enemies. Research plots were established at Texas AgriLife research center at Weslaco, Texas in fall 2007 and spring 2008 seasons, and peppers were used as a model crop. Plots were sprayed with novaluron, abamectin, spinetoram, lambda-cyhalothrin and water as treatments according to leafminer infestation; insecticide efficacy was monitored by collecting leaves and infested foliage. Plant phenology was also monitored. Novaluron was the most effective insecticide and lambda-cyhalothrin showed resurgence in leafminer density in fall 2007 and no reduction in spring 2008. Other compounds varied in efficacy. Novaluron showed the least number of parasitoids per leafminer larva and the lowest parasitoid diversity index among treatments followed by spinetoram. *Liriomyza trifolii* (Burgess) was the sole leafminer species on peppers, and 19 parasitoid species were found associated with this leafminer. Application of these insecticides for management of leafminers with conservation of natural enemies is discussed.

## Introduction

*Liriomyza* (Diptera: Agromyzidae) leafminer adults and larvae can directly damage plants as follows: The adult female punctures plant tissue with its ovipositor, which can insert an egg and/or create an open wound to allow the adult to feed on the exudate (Dimetry 1971; Bethke and Parella 1985; Parella 1987). Neonate larvae start tunneling primarily in the palisade mesophyll and complete the feeding period during four instars within the leaf (Dimetry 1971; Parella et al. 1985). Larval tunneling damages the plant by reducing the plant's photosynthetic capacity, causing leaf abscission (Parrella 1987), death of young seedlings (Elmore and Ranney 1954), reduced aesthetics (Parrella et al. 1985), transmission of diseases (Zitter and Tsai 1977) and decline of crop yields (Wolfenbarger 1954; Ledieu and Heyler 1985; Weintraub and Horowitz 1995).

*Liriomyza* leafminers feed on plants in many families including Umbelliferae, Solanaceae, Malvaceae, Liliaceae, Leguminosae, Curcurbitaceae, Compositae, and Chenopodiaceae (Stegmaier 1966). In South Texas, *Liriomyza* attacks many agricultural crops including celery, tomato, melon, cucumber, watermelon, cotton, and, in particular, pepper. The principal method for the control of *Liriomyza* leafminers in the Lower Rio Grande Valley of Texas is application of various insecticides with various modes of action in a variety of formulations. These include pyrethroids, avermectins, cyromazine, methamidophos, spinosyn and neem-based insecticides (azadirachtin) (Schuster and Everett 1983; Trumble 1984; Hara 1986; Keil and Parrella 1990; Weintraub and Horowitz 1997; Mujica et al. 2000; Civelek and Weintraub 2003; Ferguson 2004). Several insecticide applications each season are applied in the Lower Rio Grande Valley of Texas to control *Liriomyza* (Hernandez et al. 2010; Liu unpublished data).

Extensive use of insecticides has resulted in development of resistance of many species of *Liriomyza* to many active ingredients such as those found in pyrethroids, methamidophos (Keil and Parrella 1990), avermectins, cyromazine, and spinosyn (Keil and Parrella 1990; Ferguson 2004). In addition, insecticide applications have negatively affected survival and fitness of natural enemies of *Liriomyza* (Trumble and Toscano 1983; Darvas and Polgar 1998; Weintraub 2001; Prijono et al. 2004; Bjorksten and Robinson 2005; Hidrayani et al. 2005; Kaspi and Parrella 2005; Tran et al. 2005; Hossain and Poehling 2006).

Some of the commonly used insecticides in the Lower Rio Grande Valley of Texas are novaluron, spinetoram, abamectin and lambda-cyhalothrin. Novaluron (1-[chloro-4-(1, 1, 2-trifluoro-methoxyethoxy) phenyl]-3-(2, 6-difluorobenzoyl) is a benzoylphenyl urea that inhibits chitin development (Ishaaya and Casida 1974), causes unsuccessful endocuticular deposition and disrupts molting (Mulder and Gijswijk 1973). It acts primarily by ingestion, but also has some contact and translaminar activities (Ishaaya et al. 2003). Spinetoram (mixture of spinosyn A and spinosyn D) is a nicotinic acetylcholine receptor agonist. This insecticide belongs to the family of spinosyns and causes disruption of the central nervous system by hyperexcitation (Sparks et al. 2001). Abamectin (avermectin B1a and avermectin B1b) is a macrocyclic lactone, and targets the nervous system as a chloride channel activator. Avermectins bind to ligand-gated chloride channels (e.g., GABA or glutamate) where they cause inhibition of nerve firing (Bloomquist 2003; Brown 2005). Lambdacyhalothrin ([1a(S*),3a(Z)]-(±)-cyano-(3-phenoxyphenyl)methyl-3-(2-chloro-3,3,3-trifluoro-1-propenyl)-2,2-dimethylcyclopropanecarboxylate) is a pyrethroid insecticide, which acts on the nervous system as a sodium channel modulator. This axonic poison prevents sodium channels from closing, causing over-excitation and subsequent paralysis (Narahashi 1971).

**Table 1.**
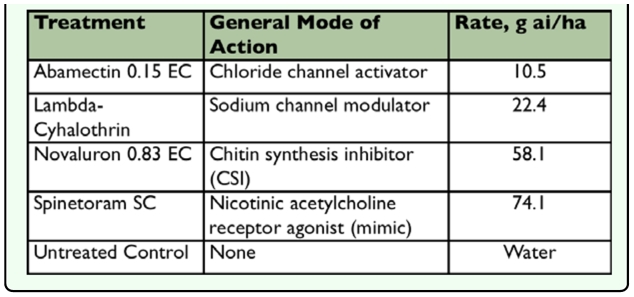
Treatments, general mode of action and application rates.

The objective of this research was to evaluate the different insecticides commonly used for control of *Liriomyza*, their efficacy in controlling leafminers in the field, and their effects on the natural enemies of leafminers.

## Materials and Methods

### Plot design

Field plots were established at the Research Farm, Texas AgriLife Research Center at Weslaco, Texas (26° 09′33.01″ N, 97° 57′32.67″ W) in fall 2007 and spring 2008 growing seasons. Plots (9.1 m × 20.4 m) were arranged in a randomized complete block design. Two rows of sorghum were planted as windbreak between the plots to avoid insecticide drift among the treatments. The pepper variety used was Jalape***ñ***o M (Seminis Vegetable Seeds, Oxnard, CA) and the seeds were planted inside a greenhouse with a management program similar to the one used by farmers. After the plants reached the fourth true leaf stage, they were transplanted in a single row in the plots at a spacing of 12 cm. The plots were drip-irrigated and fertilized and chemical insecticide was applied in the experimental treatments (see below) along with an untreated control.

### Treatments

In fall 2007 and spring 2008, four insecticides (abamectin, novaluron, spinetoram and lambda-cyhalothrin) were applied to peppers as treatments and water was used as the control (total of 5 treatments). Treatments were replicated three times (n=15 plots) in fall 2007 and four times (n=20 plots) in spring 2008. Plots were treated with insecticides using the company recommended rates (Table 1).

In order to avoid insecticide drift, wind was monitored before application with a hand wind speed sensor and no application was made if the wind speed was >4.5 kph. The chemical sprayer was calibrated to deliver 188 liters per ha, with 13 nozzles separated at 51 cm (TeeJet 8002VS) delivering 372 ml/30 sec/nozzle. Applications were made according to leafminer infestation density of 3 mines per leaf in the middle of the plant. In fall 2007, two applications were made, one on 26 October 2007, and a second one on 25 November 2007. In spring 2008 the leafminer infestation was less severe requiring only one application on 8 April 2008.

### Sampling methods

Two methods were used to sample the plots. For method one, stratified leaf sampling was used to determine insecticide efficacy in controlling leafminers. Leaves were randomly selected from each stratum at the bottom (10), median (10), and top (10) portion of the plant in each plot (15 plots, 450 leaves) bi-weekly. The samples were taken from interior plants 2m from the border in each plot to minimize any “edge effect.” This spacing was in addition to a row of sorghum separating the plots (1 m). The 10 leaves from each stratum were placed in 1 gal zipper bags. The bags were labeled according to treatment, transported to the laboratory, and processed for emergence of specimens. The leaves were examined and occupied mines, empty mines and larvae were recorded.

Leaves were separated on a paper towel, then placed back into the bag with a spacer cup in the middle to prevent the top of the bag from touching the leaves. The bags were closed, and cotton balls were placed at the closing corner of the zipper bag to allow air diffusion. The bags were held in an insectary at 28° C, at a photoperiod of 11:13 (L:D) h. Bag interiors were checked for adult emergence every 10 days (30 days total) and leafminers and parasitoids were collected, and identified to species. The leafminer density per treatment through time was calculated. In addition, insecticide efficacy compared to the untreated control was also calculated.

For method two, infested foliage sampling was used to compute the number of parasitoids per leafminer larva, and compare the parasitoid species diversity among the different treatments to evaluate the effects of treatments on natural enemies. Infested foliage was identified and ten mined-leaves with larvae were collected from each plot at 2-week intervals. The leaves were placed in plastic zipper bags and processed as above. The number of larvae, mines, emerged leafminers, and emerged parasitoids were recorded. The leafminer species attacking pepper, parasitoid species composition, and the biodiversity among treatments was also determined.

Parasitoids per leafminer larva (mean number of parasitoids/mean number of leafminer larvae) were calculated in order to monitor the effects of insecticides on the parasitoid larvae. This ratio was calculated instead of percent parasitism to avoid numerical biases from gregarious parasitoid species.

### Data analysis

Analysis of variance (ANOVA) using GLM procedure (alpha=0.1) was used to compare leafminer density among treatments on each sampling date. Insecticidal effects were calculated using Abbott's formula (density of control — density of treatment / density of control) and analysis of variance (ANOVA) (GLM procedure; alpha =0.1) were used to compare insecticide efficacy per treatment in the fall 2007 and spring 2008 samples (SAS institute 2000). Ratios of parasitoids per leafminer larva (i.e., mean number of parasitoids/mean number of leafminer larvae) were calculated to determine the effects of insecticides on parasitoid species attacking larvae. Analysis of variance (ANOVA) (GLM procedure alpha= 0.1) was used to compare parasitoid(s) per leafminer larva among different treatments on each date sampled. Furthermore, Shannon-Wiener diversity index,


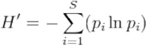


was calculated per treatment to analyze the effects of different insecticides on parasitoid diversity. Analysis of variance (ANOVA) (GLM procedure alpha 0.1) was used to compare *H'* among treatments (SAS institute 2000).

## Results

**Table 2.**
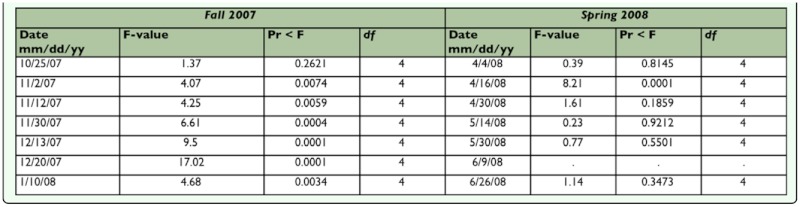
Leafminer density statistics values for fall 2007 and spring 2008.

### Evaluation of insecticides for leafminer management

**Figure 1.**
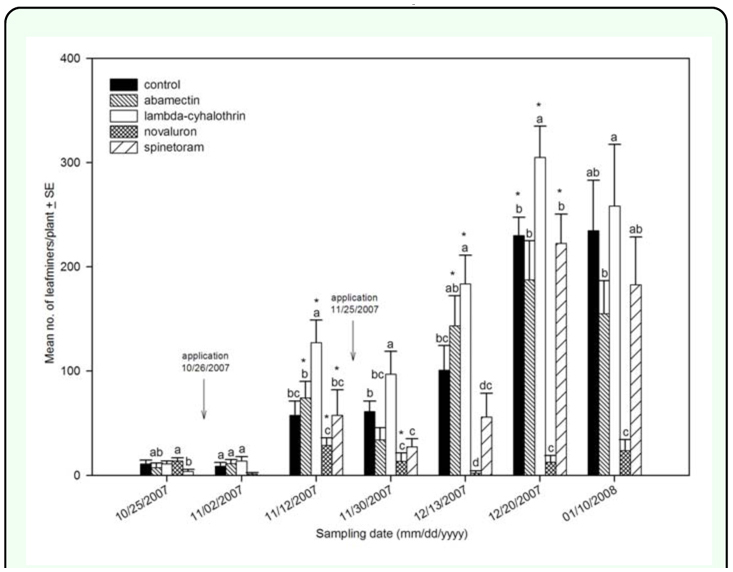
Leafminer larval density per plant in fall 2007. Arrows represent application date. Different letters represent statistical differences among treatments. Star (*) represents significant statistical differences from previous sample date within treatments. High quality figures are available online.

**Figure 2.**
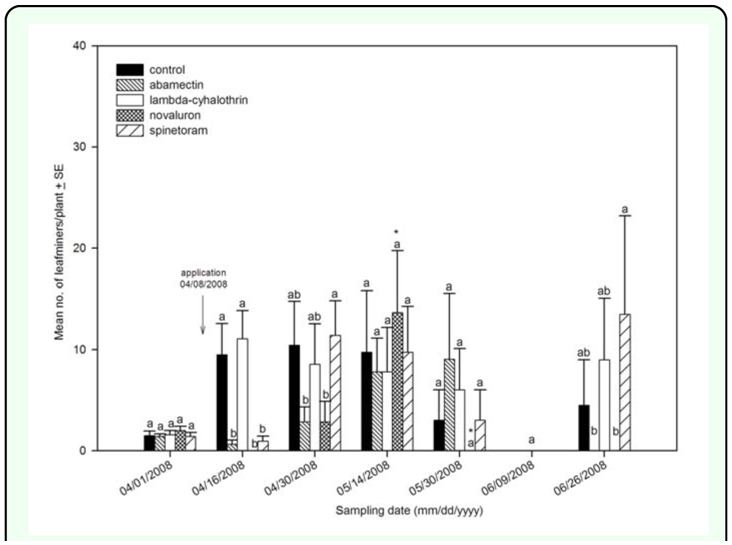
Leafminer larval density per plant in spring 2008. Arrows represent application date. Different letters represent statistical differences among treatments. Star (*) represents significant statistical differences from previous sample date within treatments. High quality figures are available online.

In fall 2007, treatment with novaluron resulted in lower leafminer larval density per plant compared to the control at 7 and 17 days after the first application (Figure 1, Table 2). After a second application, a similar trend was measured on 30 November and 13 December 2007 when novaluron had the lowest larvae per plant among treatments. Novaluron was the only insecticide producing significantly lower density of larval leafminers than the control at 25 and 35 days after the second application. No statistically significant differences in density between the novaluron treatment and the control were measured in the spring 2008 experiment, except at the first evaluation on 16 April (Figure 2, Table 2).

Treatment with spinetoram resulted in lower leafminer larval density per plant than the control 7 d from first application in fall 2007. Similarly, the treatment with spinetoram produced lower larval density per plant than in either the control abamectin, or lambdacyhalothrin treatments after the second application on 30 November 2007 and 13 December 2007. Furthermore, larval density in the spinetoram treated plants increased from 13 December 2007 to 20 December 2007 (*F* = 14.16; *df* = 7, 3; *P* < 0.0001). The plants treated with spinetoram reduced leafminer density; however density increased in the following samples compared to novaluron (Figure 1, Table 2). The plants treated with spinetoram also had lower leafminer density compared to the control after the first application on 8 April 2008. No statistically significant differences were shown by subsequent samplings (Figure 2, Table 2).

Treatment with abamectin had no statistically significant effect on densities of leafminer larvae relative to the untreated control in the fall of 2007. In addition, leafminer densities increased on plants treated with abamectin (*F* = 12.17; *df* = 7, 3; *P* < 0.0001) from sample day 30 November to sample day 13 December 2007 (Figure 1, Table 2). In spring 2008, after the first application on 8 April, the plants treated with abamectin had lower leafminer density than those in the untreated control. Leafminer density on the plants treated with abamectin was not statistically different from the control beginning 22 days after application through the end of the experiment (Figure 2, Table 2).

In fall of 2007, plants treated with lambdacyhalothrin not show statistically significant effects in comparison to the untreated control seven days after the first application. Subsequently, densities of *Liriomyza* on plants treated with lambda-cyhalothrin were significantly higher than those in all treatments including the control 17 days after the first application. The leafminer density on the plants treated with lambda-cyhalothrin was statistically higher than those in the untreated control on 30 November and 13 December 2007 after the second application. In addition, an increase in leafminer density was recorded in the lambda-cyhalothrin treatment from 30 November to 13 December 2007 (*F* = 17.56; *df* = 7, 3; *P* < 0.0001). On 20 December 2007, the plants treated with lambda-cyhalothrin had a higher density than the untreated control, but were not significantly different on 10 January 2008 (Figure 1, Table 2). In spring 2008, lambda-cyhalothrin did not have an effect on leafminer density compared to the control (Figure 2, Table 2).

**Table 3.**
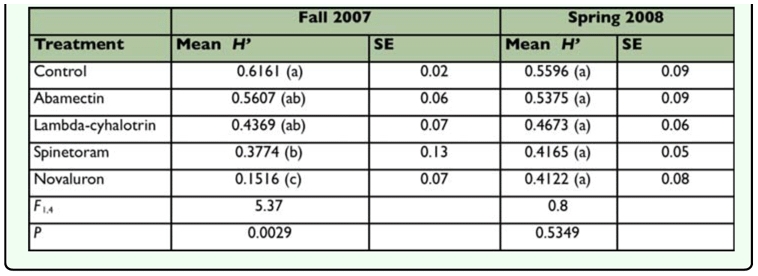
Shannon-Wiener diversity index for fall 2007 and spring 2008.

**Figure 3.**
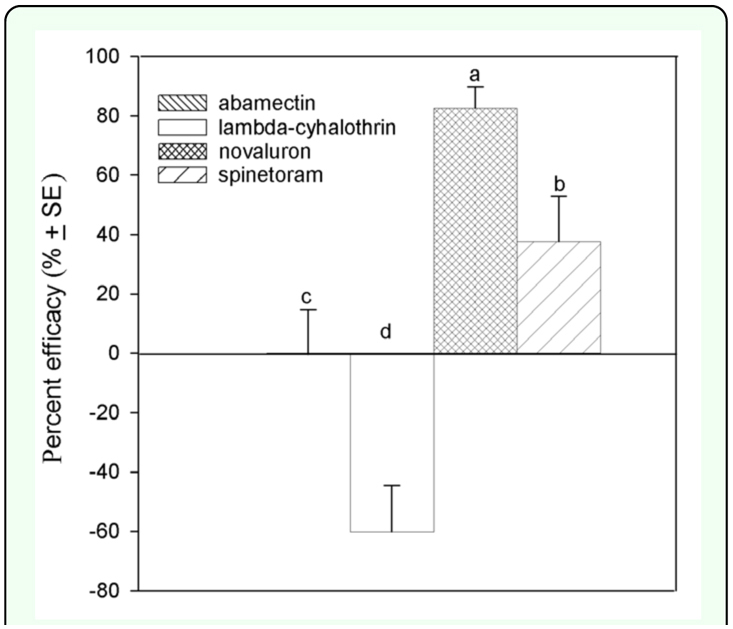
Percent efficacy compared to control in fall 2007. Different letters represent significant statistical differences among treatments. High quality figures are available online.

**Figure 4.**
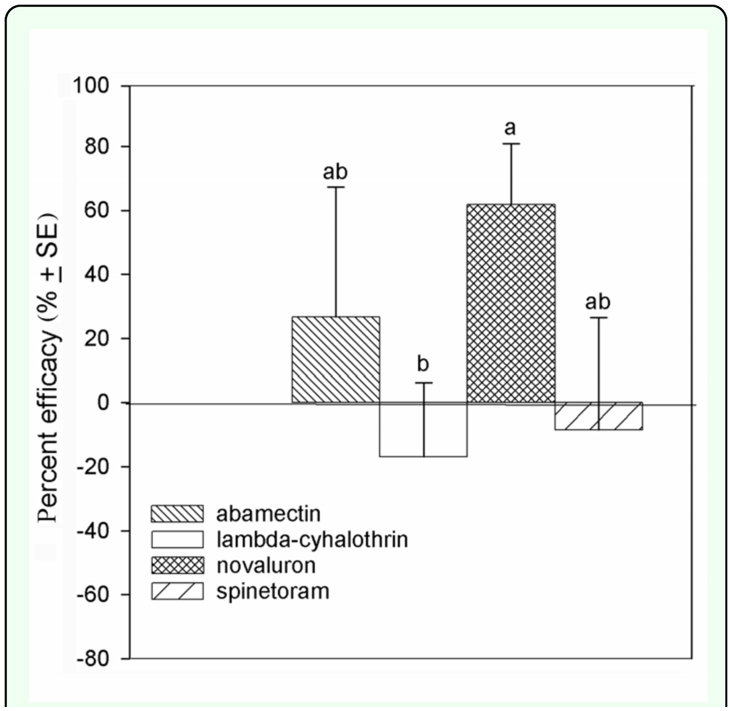
Percent efficacy compared to control in spring 2008. Different letters represent significant statistical differences among treatments. High quality figures are available online.

Based on calculations of total insecticide efficacy, in fall 2007, the most effective insecticide was novaluron followed by spinetoram (Figure 3). No effect on leafminers was measured following treatment with abamectin., whereas plants treated with lambda-cyhalothrin had increased leafminer density (*F* = 19.11; *df* = 3, 3; *P* < 0.0001) (Figure 3). In spring 2008, plants treated with novaluron had the lowest leafminer density, which was significantly lower than on those treated with lambda-cyhalothrin (*F* = 1.38; *df* = 3, 3; *P* < 0.2790) (Figure 4).

### Insecticide effects on natural enemies

**Figure 5.**
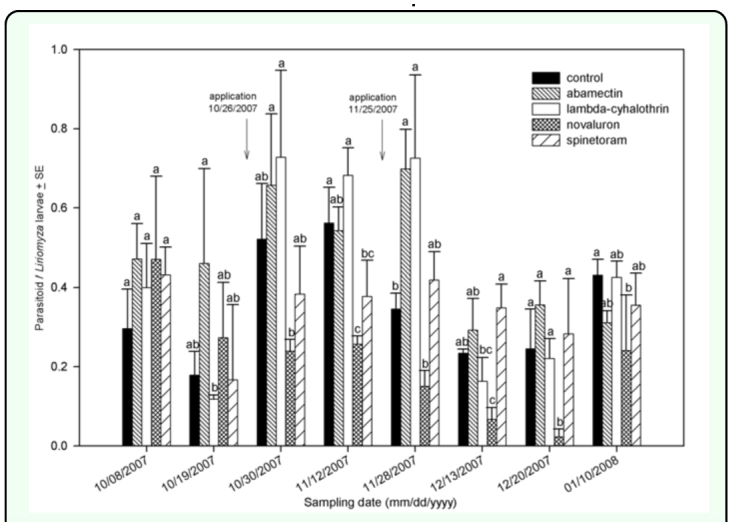
Ratio of parasitoids per leafminer larva in fall 2007. Arrows represent application date. Different letters represent significant statistical differences among treatments. High quality figures are available online.

**Figure 6.**
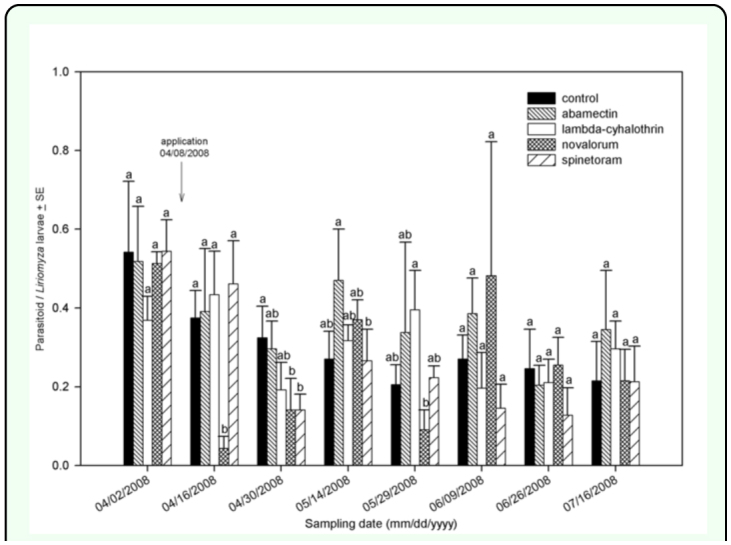
Ratio of parasitoids per leafminer larva in spring 2008. Arrows represent application date. Different letters represent significant statistical differences among treatments. High quality figures are available online.

Effects of insecticides on ratios of parasitoids to leafminer larvae were mixed and dependent on insecticide and sampling date (Figure 5). In fall 2007, the plants treated with novaluron and spinetoram had significantly lower densities of parasitoids per leafminer larva ratios than those in the untreated control 16 days after the first application (12 November 2007). The plants treated with abamectin and lambda-cyhalothrin did not show differences from the untreated control (*F* = 6.38; *df* = 4, 3; *P* < 0.0081) (Figure 5). The plants treated with novaluron showed a lower parasitoid/leafminer larva ratio after the second application on 13 December 2007 (*F*=3.64; *df=*4, 3; *P*<0.0442) 20 December 2007 (*F*=4.48; *df*=4, 3; *P*<.0.0247), and 10 January 2008 (*F*=1.20; *dj*=4, 3; *P*<0.3697). In spring 2008, the plants treated with novaluron had a lower parasitoid/leafminer ratio 8 days after the first application (16 April 2008) (*F*=2.65; *df*=4, 3; *P*<0.0747) (Figure 6). Twelve days after application (30 April 2008), the plants treated with spinetoram and novaluron had a lower parasitoid/leafminer larva ratio (*F*=1.60; *dj*=4, 3; *P*<0.2245). In fall 2007, the untreated plants had the highest Shannon-Wiener diversity index (*H'*) (Table 3). In addition, spinetoram and novaluron were the only two insecticides with statistically lower indices compared to the untreated control. Novaluron had the lowest diversity index in fall 2007 (*F*=5.37; *df*=4, 3; *P*<0.0029). In spring 2008, the *H'* in the untreated control was numerically higher than other treatments (Table 3); however no significant differences were found among treatments (*F*=0.80; *df*=4, 3; *P*<0.5349).

### *Liriomyza* and parasitoid species composition

The *Liriomyza* species composition in the fall of 2007 was found to be 454 specimens of *L. trifolii* (Burgess), one *L. sativae* Blanchard, and 33 specimens were unidentifiable due to the lack of useful morphological characters. In spring 2008, 144 specimens were identified as *L. trifolii* and 23 specimens were unidentifiable. *Liriomyza sativae* was not found in spring 2008.

**Table 4.**
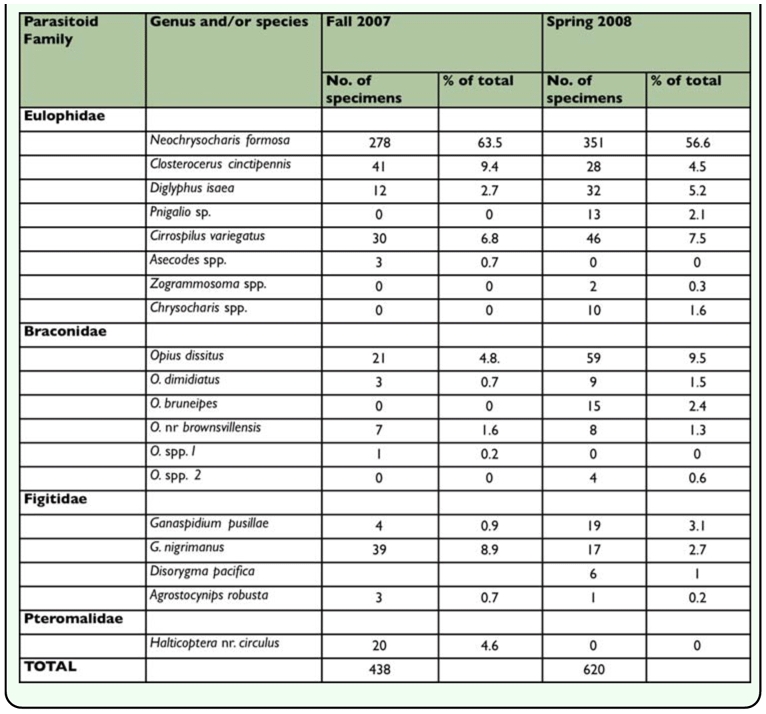
Parasitoid species composition in the fall 2007 and spring 2008 from leafminer infested leaves.

A total of 438 parasitoid specimens representing 13 species were collected in fall 2007 (Table 4). These included *Neochrysocharis formosa* (Westwood) (Hymenoptera: Eulophidae) (278 individuals collected), *Closterocerus cinctipennis* Ashmead ( (41), *Diglyphus isaea* (Walker) (12), *Cirrospilus* spp. (30), and *Asecodes* spp. (3) from the Eulophidae family, and *Opius dissitus* Muesebeck (21), *O. dimidiatus* (Ashmead) (3), *O.* nr *browsvillensis* (7), and *Opius* spp *1* (1), from the Braconidae family. In addition, *Ganaspidium pusillae* Weld (4), *G. nigrimanus* (Kieffer) (39), and *Agrostocynips robusta* (Ashmead) (3) were identified from from the family Figitidae, and *Halticoptera circulus* (Walker) (20) from the family Pteromalidae.

A total of 620 parasitoid specimens representing 16 species were collected in spring of 2008 (Table 4). These included *N. formosa* (351), *C. cinctipennis* (28), *D. isaea* (32), *Pnigalio* spp. (13), *Cirrospilus* *variegates* (46), *Zogrammosoma* spp. (2), *Chrysocharis* spp (10), from the family Eulophidae, and *O. dissitus* (59), *O. dimidiatus* (9), *O.* nr *browsvillensis* (8), *O. bruneipes* (15), and *Opius* spp *2* (4), from the family Braconidae. In addition, *G. pusillae* (19), *G. nigrimanus* (17), *A. robusta* (1), and *Disorygma pacifica* (Yoshimoto) (6) were identified from the family Figitidae.

## Discussion

The main difference between the fall 2007 and the spring 2008 trials was in leafminer density, which reached 300 leafminers per plant in fall 2007 in contrast to ∼15 leafminers per plant in spring 2008. This was mainly because crop production, climate and population dynamics of the pests and parasitoids in this area also differ between spring and fall. Novaluron was effective against leafminers in both trials, whereas treatment with lambda-cyhalothrin increased (fall 2007) or had no measureable effect (spring 2008) on leafminer density. Spinetoram was also effective against leafminers, whereas efficacy of abamectin varied in both seasons. Novaluron was the most effective insecticide as shown by low leafminer densities in both fall 2007 and spring 2008. At this time, resistance to novaluron has not been documented in populations of *Liriomyza* in South Texas.

Although resistance of *Liriomyza* species to spinosad has been documented (Ferguson 2004), spinetoram was effective in this field evaluation and reduced *Liriomyza* densities after applications in fall 2007 and spring 2008. However, its efficacy was lower than that of novaluron. In fall 2007, leafminer density in spinetoram- treated plots increased from 13 to 20 December 2007 and in spring 2008, leafminer density in the spinetoram treatment was higher compared to novaluron on 30 April 2008. This lower efficacy may be influenced by the effects of spinetoram on natural enemies, or lower plant residues for control of emerging leafminers.

Mixed results were measured in tests with abamectin. In fall 2007, overall efficacy of abamectin was nil. However, in spring 2008 when leafminer densities were lower, abamectin reduced leafminer density after application and appeared effective. *Liriomyza* resistance to abamectin has been documented (Ferguson 2004). Abamectin is a widely used insecticide in South Texas primarily for the control of mites and to a lesser extent for the control of *Liriomyza* leafminers. More detailed studies need to be conducted to determine the status of *Liriomyza* vis à vis abamectin resistance and efficacy in the Lower Rio Grande Valley of Texas.

In tests with lambda- cyhalothrin, densities of larval *Liriomyza* were either increased or unchanged relative to the controls. In addition to negative effects of lambda- cyhalothrin on natural enemies (e.g., lethal, sublethal, repellant), a possible explanation for this finding (and the low efficacy of this insecticide) may be resistance to pyrethroids in *L. trifolii..* Several agricultural pests (including leafminers in the Lower Rio Grande Valley) are resistant to pyrethroids, including lambda- cyhalothrin. Thus, additional studies on the resistance to lambdacyhalothrin in *L. trifolii* from the Lower Rio Grande Valley of Texas may help to explain the increase of *Liriomyza* density in this area.

Based on the field data, novaluron had negative effects on immature parasitoid stages. Novaluron toxicity may directly or indirectly affect parasitoids because it was effective against the *Liriomyza* leafminers. Although no research has been done on this insecticide's effects on natural enemies of *Liriomyza* leafminers, Bastos et al. (2006) tested its effect on *Trichogramma pretiosum* Riley, parasitizing the Mediterranean flour moth [*Ephestia kuehniella* (Zeller)], and found that novaluron did not affect the adult parasitoids, but interfered with development of immature stages within the host egg. Spinetoram also negatively affects parasitoid immature stages, and previous research has shown that this insecticide is harmful to natural enemies (Hossain and Poehling 2006). However, our data suggest that it was no more toxic to the parasitoid complex compared to the untreated control. In this regard, more laboratory or field experiments should be conducted to assess the toxicity of spinetoram to parasitoids of leafminers in South Texas. In results from a separate study, insecticides tested here were shown to have differing effects on the adult parasitoids. For example, novaluron was the least toxic, whereas abamectin had significant mortality and spinetoram was the most toxic (Hernández and Liu, unpublished results). Effects of lambda-cyhalothrin on adults differed between species: it was toxic to *G. nigrimanus* (a larval-pupal parasitoid of *Liriomyza*) but had no effect on *N. formosa. Neochrysocharis formosa* was the most abundant parasitoid in the survey. *N. formosa* is a larval parasitoid and has a close association with larval leafminers. This association could potentially have resulted in an evolved parasitoid tolerance to the compound. *N. formosa* represented 60% of the total parasitoids collected and a developed tolerance of *N. formosa* to lambda-cyhalothrin could explain the lack of a difference in parasitoid abundance and diversity between the control and lambda-cyhalothrin in the research plots. Natural enemy diversity is an important factor in evaluating conservation biological control (Altieri 1999, Landis et al. 2000, Cai et al. 2007).

*Liriomyza trifolii* damaged Jalape***ñ***o M in the insecticide plots. It accounted for 99 percent of the identified specimens. The parasitoid per leafminer ratio was much higher in spring 2008 than fall 2007. The higher abundance of parasitoids in spring 2008, potentially contributed to the lower densities of leafminers in the experimental plots observed during this season.

In summary, this study shows that a diverse group of hymenopterous parasitoids is associated with *Liriomyza* in the Lower Rio Grande Valley of Texas and they, at times, play a role in the economic suppression of infestations. We expect density dependant responses and the relative searching abilities of these parasitoids may also be affecting how this parasitoid complex responds to various treatments. Partitioning the effects of insecticides and parasitoids on leafminer density is challenging. Leafminer densities in the fall of 2007 triggered two insecticide treatments using our action level of 3 mines per leaf in the middle of the plant; leafminer densities increased after treatment and on the last fall sampling date were economically damaging with only the novaluron treatment providing control. Leafminer densities in the spring of 2008 triggered one insecticide treatment, however, leafminer densities on subsequent spring sampling dates did not increase as they had in the fall to economically damaging levels in any treatment, including the control. These factors should be considered when interpreting these data. For example, the low leafminer densities and the low parasitoid diversity in the fall of 2007 novaluron treatment may be due to a direct effect of the insecticide on *Liriomyza*, which reduces parasitoid density through lack of prey, or a combined toxicity to *Liriomyza* and to the parasitoids, or some combination of both. This issue is less relevant in the spring of 2008 because economic densities of leafminers did not continue as they had in the fall to actually cause economic damage. Further work is needed in this area.

The leafminer species *Liriomyza trifolii* exhibits such a long history of insecticide resistance that it is doubtful that the use of insecticides will provide sustainable control, even if new, effective insecticides with novel modes of action are found. We recommend avoidance of insecticide application whenever possible to conserve natural enemies. If insecticide application is required, a one time application of novaluron should be used to control *L. trifolii* outbreaks. Use of insecticides must be integrated with biological controls. If an infestation requires more applications, an insecticide rotation program should be planned to avoid/delay development of resistance. Lambda-cyhalothrin, which is ineffective in controlling *L. trifolii*, should be avoided.
